# P-1727. Impact of Four-Day Automatic-Stop Orders on the Duration of Antibiotic Treatment

**DOI:** 10.1093/ofid/ofae631.1891

**Published:** 2025-01-29

**Authors:** Aaron Oliver, Andrea Quinn, Amanda Ries, Erin Weslander, Katarzyna Malkowicz

**Affiliations:** Northwestern Medicine, Chicago, Illinois; Northwestern Medicine Palos Hospital, Palos Heights, Illinois; Northwestern Medicine, Chicago, Illinois; Northwestern Memorial Hospital, Chicago, Illinois; Northwestern Medicine, Chicago, Illinois

## Abstract

**Background:**

Implementation of automatic antibiotic stop orders is an antimicrobial stewardship intervention that encourages timely antibiotic order re-evaluation for potential discontinuation, spectrum de-escalation, transition to oral therapy, or spectrum escalation if warranted based on emerging diagnostic results. This pre-post study was designed to evaluate the impact of a four-day automatic-stop order on antibacterial orders for patients admitted at a single community hospital site.

**Figure:**
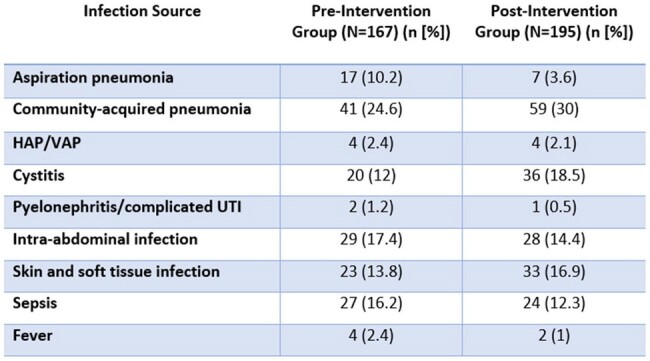

**Methods:**

A single-center, retrospective pre-post study was conducted to compare outcomes following the re-introduction of a four-day automatic-stop order on antibacterial orders. The primary outcomes were hospital length of stay and median duration of antibiotic therapy. Secondary outcomes included hospital readmission within 30 days and the occurrence of C. difficile infections within 30 days. Data was compared between 2 non-consecutive weeks in the pre-implementation of the automatic-stop order policy with data from 2 non-consecutive weeks in the post-implementation of the automatic-stop order policy to ensure prescriber variability and prescribing practices. Patients admitted for fewer than 4 days, or who had orders with indications for meningitis, bacteremia, prosthetic material infection, bone and joint infections, endocarditis, *C. difficile* treatment, prophylaxis, or documented uncontrolled source were excluded to align with exclusions in the policy.

**Figure:**
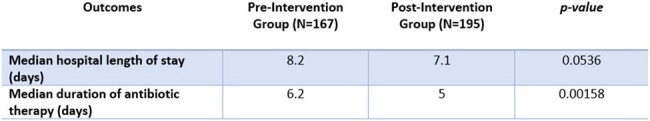

**Results:**

The study included 362 patients in total, 167 in the pre-implementation group and 195 in the post-implementation group. The median hospital length of stay was 8.2 days in the pre-implementation group and 7.1 days in the post-implementation group (p=0.536). The median total treatment duration of antibiotic therapy was 6.2 days in the pre-implementation group and 5 days in the post-implementation group (p=0.02). Incidence of c. difficile was 3% in the pre-implementation group, compared to 1% in the post-implementation group (p=0.256).

**Figure:**
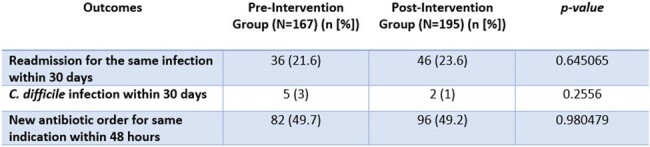

**Conclusion:**

An antimicrobial automatic stop policy of four days was associated with decreased antimicrobial days of therapy per patient, without differences in hospital length of stay. There were no safety concerns identified with this intervention.

**Disclosures:**

**All Authors**: No reported disclosures

